# Acute Effects of High-Intensity Interval Training on Brain-Derived Neurotrophic Factor, Cortisol and Working Memory in Physical Education College Students

**DOI:** 10.3390/ijerph17218216

**Published:** 2020-11-06

**Authors:** Inmaculada C. Martínez-Díaz, María C. Escobar-Muñoz, Luis Carrasco

**Affiliations:** 1 Department of Physical Education and Sport, University of Seville, E-41013 Seville, Spain; martinezdiaz@us.es; 2 Virgen del Rocío University Hospital, E-41013 Seville, Spain; mcarmenescobar@hotmail.com

**Keywords:** exercise, HIIT, neurotrophins, stress responses, cognition, executive functions

## Abstract

High-intensity interval training (HIIT) is considered one of the most effective methods for improving cardiorespiratory and metabolic functions. However, it is necessary to clarify their effects on neurophysiological responses and coginitive functioning. Thus, this study aimed to determine the effects of an acute bout of HIIT on neurocognitive and stress-related biomarkers and their association with working memory (WM) capacity in healthy young adults. Twenty-five male college students performed a single bout of HIIT consisting of 10 × 1 min of cycling at their VO_2_ peak power output. Plasma Brain-Derived Neurotrophic Factor (BDNF) and cortisol (CORT) levels, and WM (Digit Span Test (DST)), were assessed pre-, post- and 30 min post-intervention. Significant post-exercise increases in circulating BDNF and CORT levels were observed coinciding with the highest DST performance; however, no statistical associations were found between cognitive and neurophysiological variables. Moreover, DST scores obtained 30 min after exercise remained higher than those assessed at pre-exercise. In conclusion, the stress induced by a single bout of HIIT induces a remarkable response of BDNF and CORT boosting WM capacity in healthy young males. Future research should clarify the association between cognitive and neurobiological markers during intense exercise stimulation.

## 1. Introduction

High-intensity interval training (HIIT), which involves alternating bouts of intensive exercise with low-intensity or passive recovery periods [1,2], has been considered one of the most effective methods for improving cardiorespiratory and metabolic functions [3]. Thus, the use of HIIT to enhance health-related outcomes has recently generated new interest [4,5]. However, little information about the effects of HIIT on mental health and other related-psychological outcomes exists [6,7]. Several investigations have focused on the effects of HIIT on brain function, but their results are sometimes uncertain and contradictory [8,9,10,11,12]. Indeed, the current evidence showed a positive impact of HIIT on the brain, specifically in neurotrophin expression and function [13].

It is known that brain-derived neurotrophic factor (BDNF), a protein of 252 amino acids, plays a key role to maintain or improve several brain functions such as neuronal protection and survival, neurite expression, axonal and dendritic growth and remodelling, neuronal differentiation and synaptic plasticity [14]. The response of BDNF to acute exercise has been investigated by several authors using different exercise protocols and consequently, reporting different results (from the lack of response to increases between 11.7 and 410.0% regarding the basal levels). Moderate or high-intensity exercises induce greater increases of BDNF [15,16,17,18]; however, it is important to indicate that the protocols used in some of these investigations do not make them conclusive, to which must be added the existence of other studies in which no significant increases in BDNF levels have been observed after efforts to this nature [19,20,21].

In addition, and according to previous findings [22], this neurotrophin could also be involved in learning and memory processes. Considering this hypothesis, different studies were performed trying to determine if the post-exercise increase in BDNF concentration could boost executive functions (EF). EF are a set of basic mental processes that support planning, reasoning, or problem-solving [23] among which working memory (WM) is one of the most important functions (WM involves holding information in mind and mentally working with it and also enables us to bring conceptual knowledge and not just perceptual input to bear on our decisions). In fact, several studies showed improvements in EF and WM capacities after a single bout of exercise, including HIIT [24,25,26,27]. However, others have found a lack of association between exercise-related BDNF responses and WM [28,29].

On the other hand, a well-known exercise stress response is obviously related to the reactivity of the hypothalamic–pituitary–adrenal (HPA) axis. Cortisol (CORT), a glucocorticoid hormone produced by the adrenal cortex, is a corticosteroid released in response to stress as the end product of the HPA axis [30]. The CORT responses to stress induced by an acute bout of exercise are well documented [31] and its increases in the bloodstream are related to the magnitude of exercise-induced stress; so, high-intensity and long-duration exercise induces the greatest CORT releases. Moreover, previous studies have reported an inverted U-shaped relationship between CORT and EF [32] with moderate levels of this glucocorticoid being positively associated with improvements in EF [33]. Nevertheless, more recent studies have shown an inverse relationship between exercise-derived CORT and EF [34].

Taking into account all these considerations, certain aspects should be addressed in determining the potential role of acute exercise improving cognitive functioning. First, the evidence is beginning to emerge that aerobic exercise might also be beneficial for such functioning in young adulthood, despite executive functioning peaking developmentally in that age group [35]. On the other hand, a number of studies have used aerobic exercise as a stimulus for EF; however, it is necessary to clarify the acute effects of high-intensity exercise on WM and to determine a possible role of BDNF and CORT.

Thus, the present study aimed to determine the effect of an acute bout of HIIT on WM in healthy young adults focusing on the underlying neurophysiological and stress markers. We hypothesized that stress induced by an acute bout of HIIT would boost WM while the circulating levels of BDNF and CORT remain elevated, which would mean a different neurophysiological pattern from that previously established.

## 2. Materials and Methods

### 2.1. Participants

Twenty-five male physical education college students (mean ± sd, age: 21.7 ± 2.1 years; height: 1.77 ± 0.06 m; weight: 72.6 ± 8.4 kg; body mass index, BMI: 23.1 ± 1.4 Kg/m^2^) were selected to participate in this study. The inclusion criteria were as follows: a) age between 18 and 25 years; b) good general health with no diseases expected to interfere with the study; and c) no tobacco, alcohol, and other drug use. On the other hand, the exclusion criteria were: DSM-5 diagnostic criteria for neurocognitive disorders within the last 12 months, bilingualism or multilingualism, to play musical intruments or to study music daily, imminent exams, any psychiatric or neurological disorders, cardiovascular, metabolic, immunological, and endocrine diseases—especially Adisson’s and Cushing’s diseases—or medication intake that could influence CNS functioning and the study outcomes.

The calculations for sample size and power were based on BDNF responses reported by previous studies that used participants and experimental designs of similar characteristics to this study [21,36]. Taking into account the large effect sizes (ES) showed by these references (range of Cohen’s *d* = 0.63 − 1.16) the a priori sample size calculation (G*Power v.3.1) with ES = 0.64 established that a sample of 22 would be sufficient to obtain a statistical power of 0.8 (*p* < 0.05); therefore, our sample size of 25 allowed us to overcome a power of 86%.

All subjects gave their informed consent for inclusion before they participated in the study. The study was conducted in accordance with the Declaration of Helsinki [37], and the protocol was approved by the Ethics Committee of Univesity of Seville.

### 2.2. Experimental Approach

A one-group pretest/posttest quasi-experimental design was used in this study which involved two separate testing sessions with at least 5 days between them. Participants’ body composition was measured using bioelectrical impedance (TANITA BC-418MA) and a graded exercise test on a cycle ergometer (Ergoline Ergoselect 200) was also performed in the first session, in which subjects were also familiarized with the WM evaluation task. The second session consisted of (a) 15-min of sitting rest; (b) pre-intervention assessment: venous blood collection and WM test; (c) exercise-based intervention (HIIT) consisting of 10 x 1-min bouts of cycling on the same ergometer (the cadence was set at 70 rpm and the resistance—watts—was individually adjusted according to power output corresponding to the VO_2_ peak) with 1 min of passive recovery in which the participants remained seated wtih their legs resting on the pedals; (d) post-intervention assessment and (e) 30-min post-intervention evaluation using the same procedures and order of measurements used for pre-intervention. For each session (conducted between 8:00 a.m. and 10:00 a.m.), participants were instructed to avoid strenuous physical activity during the previous 24 h and to abstain from food (overnight fasting), caffeine, cacao, and alcohol 12 h before testing.

### 2.3. Exercise-Related Measurements

#### 2.3.1. VO_2_ Peak

At the first session, all participants performed a maximal incremental exercise test to determine peak VO_2_ (VO_2_ peak) on a cycle ergometer. After 5 min of cycling at 50 W, the workload was increased at a rate of 25 W/min until exhaustion (the cadence was set at 70 rpm). During the incremental test, breath-by-breath pulmonary gas-exchange data were collected (*CPX, MedGraphics*, Saint-Paul, MN, USA). Heart rate (HR) was also measured continuously by telemetry (X-Scribe, Mortara, Milwaukee, WI, USA). The VO_2_ peak was determined as the highest 20 s mean value attained before exhaustion, which was defined according to the ACSM criteria [38]. The individual power output corresponding to the VO_2_ peak (VO_2_ peak power) was recorded for the HIIT bout.

#### 2.3.2. HIIT HR and RPE Assessment

At the second session, HR was monitored continuously during HIIT by an HR monitor (Polar RCX5; Polar Electro Ltd., Kempele, Finland). Mean and maximal HR were assessed for each bout of cycling. The Borg’s 6–20 scale [39] was shown to the participants at the end of each cycling interval to assess their ratings of perceived exertion during HIIT.

### 2.4. Neurophysiological Assessment

At the second session, blood samples were taken from the participants’ antecubital vein 5 min before HIIT (pre-intervention), just after HIIT (post-intervention), and 30-min after HIIT (30-min post-intervention). Blood samples, which were collected in EDTA tubes, were immediately centrifuged for extraction of plasma (3000 rpm for 15 min at 4 °C), which was aliquoted and stored at −80 °C until analysis.

#### 2.4.1. BDNF

Plasma BDNF concentrations were analyzed in duplicate by enzyme immunoassay (ELISA) using an Abnova^TM^ kit KA0329 (Tapei City, Taiwan), with a detection range from 31.2 to 2000 pg/mL, and no detectable cross-reactivity with other neurotrophins. The intra-assay and inter-assay variations were <5% and <8%, respectively.

#### 2.4.2. CORT

Plasma CORT levels were analyzed in duplicate by ELISA using a specific kit purchased from Abnova^TM^ (KA3382; Tapei City, Taiwan), with a detection range from 30 to 230 ng/mL, and cross-reactivity with prednisolone (5.1%), corticosterone (0.3%), and progesterone-estradiol (<0.1%). The average inter-assay coefficient of variation (CV) across all assays was 8.6% and the average intra-assay CV was 6.2%.

### 2.5. WM Task

WM capacity was assessed pre, post, and 30-min post-HIIT using the Digit Span Test (DST) from the Wechsler Adult Intelligence Scale—Fourth Edition (WAIS-IV) [40]. This task consists of increasingly long sequences of random numbers that are orally presented at a rate of one digit per second to the participants, who have to repeat the sequence in two conditions: in the forward condition (DST-F), sequences of digits have to be repeated in the same order as presented; in the backward condition (DST-B), digit sequences have to be repeated in reverse order. In both conditions, the task is stopped when a subject fails to recall at least two strings of the same length or repeats the last sequence correctly (eight digits for DST-B and nine for DST-F). Thus, the DST performance is defined by three scores: DST-F score (nine points maximum), DST-B score (eight points maximum) and total DST score (DST-T) which is the sum of DST-F and DST-B scores.

### 2.6. Statistical Analysis

The data are expressed as mean ± standard deviation (sd). Kolmogorov–Sminorv and Shapiro–Wilk tests were applied to test for a normal distribution of variables. Taking into account that data were not normally distributed, non-parametric tests (Friedman and Wilcoxon signed-rank tests) were used to determine the intragroup differences between the pre- and post-intervention assessment time points. Moreover, effect size (ES) was calculated using the *r*-value proposed by Cohen [41]; thus, the ES was interpreted as trivial when r < 0.1, small: r = 0.1–0.3, medium: r = 0.3–0.5, and large: r > 0.5. A bivariate correlation was also performed using the Spearman’s rho which was set at 0.500 for a positive correlation. For all tests, a *p*-value < 0.05 was used to indicate statistical significance.

## 3. Results

### 3.1. Graded Exercise Test: refErence Data

As can be seen in Table 1, the mean time to exhaustion in the graded exercise test was 13.6 ± 1.8 min. Taking into account the protocol performed, this time was appropriate to define both HR_max_ (181.5 ± 8.4 bpm) and VO_2_ peak (45.3 ± 9.3 mL/kg/min), at which the participants developed a mean power (pVO_2_ peak) of 275.0 ± 48.9 W.

### 3.2. Cardiovascular and RPE Responses to HIIT

HR values showed a gradual increase from the first to the last repetition in the HIIT-based intervention. Maximal HR (HR_max_) rose progressively from 146.6 ± 11.6 bpm to 177.8 ±10.9 bpm; in parallel, mean HR values (HR_mean_) increased from 126.3 ± 11.5 bpm to 159.5 ± 14.2 bpm.

Data regarding RPE showed a similar tendency to that observed in HR. RPE scores increased from 11.9 ± 2.3 points at the first repetition to 18.5 ± 1.8 points at the tenth.

### 3.3. BDNF and CORT Assessment

Peripheral plasma concentrations of BDNF rose significantly from 424.66 ± 91.05 to 1271.07 ± 342.93 pg/mL (*p* < 0.001) just after intense exercise. However, they returned to the pre-exercise levels in the next 30 min (Table 2).

Regarding plasma CORT levels, they progressively increased from pre-exercise (132.33 ± 35.88 ng/mL) to 30 min after HIIT intervention (234.45 ± 109.63 ng/mL; p < 0.001) (Table 2).

### 3.4. WM Evaluation

**Figure 1:**
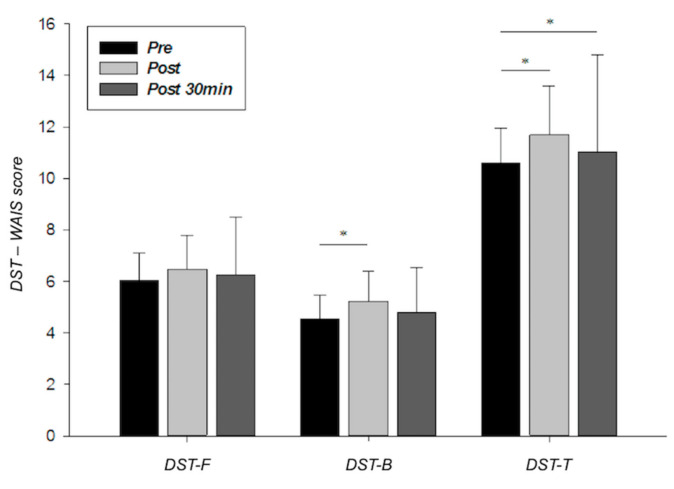
DST-F, DST-B, and DST-T scores measured at the intervention’s time-points (session 2). Error bars represent standard deviations. WAIS = Wechsler Adult Intelligence Scale; DST: Digit Span Test; DST-F = Digit Span Test in the forward condition; DST-B = Digit Span Test in the backward condition; DST-T = total Digit Spam Test score. * *p* < 0.05.

As can be observed in Figure 1, a single bout of HIIT induced increases in DST scores with results reaching statistical significance in DST-B and DST-T (5.2 ± 1.3 and 11.7 ± 1.9, respectively; *r* = 0.392–0.511). However, DST scores assessed 30 min after exercise were slightly lower than those measured just at the end of the exercise (6.2 ± 2.2, 4.8 ± 1.8, and 11.0 ± 3.8 for DST-F, DST-B, and DST-T, respectively) but they were still higher than those observed in the pre-exercise situation.

### 3.5. Relationship Between Variables

Correlation analyses did not reveal any significant relationship between variables measured at at the same assessment time-point. However, significant relationships were observed when plasma levels of BDNF and CORT were analyzed across the assessment time-points (Table 3).

## 4. Discussion

As we hypothesized, the HIIT-based intervention induced a remarkable response of BNDF and CORT, also improving WM capacity. Moreover, this bout of intense exercise provoked an important increase in both HR and RPE, reaching values near to the maximum. These results coincide in part with those provided by Rozenek et al. [42], who reported similar mean HR_max_ and HR_mean_ percentage values to those found here. However, RPE data measured in the present study were slightly higher than those previously observed by these authors.

Before addressing the impact of HIIT on the BDNF response it is necessary to highlight the remarkable interindividual differences in BDNF resting levels reported by previous studies, having established a wide range of plasma concentration (from 10.3 to 2500 pg/mL) [43]. In our study, the plasma levels of BDNF found before the HIIT (424.66 ± 91.05 pg/mL) were in the normal range previously mentioned; however, reduced interindividual differences were observed due, mainly, to the homogeneity of the sample in terms of age, body mass index, fitness level, and absence of metabolic, neurological and immunological diseases.

Taking into account the pre-exercise data, a huge increase (around 200%) in BDNF plasma levels of this neurotrophin was observed just at the end of the exercise (Table 2). However, this increase in plasma BDNF concentration could be considered moderate, since previous studies have reported increases of up to 400% after intense exercise [18,21,28,44,45]. Gustafsson et al. [15] reported increases of 398% in BDNF circulating levels after exhausting exercise but, interestingly, the analysis of BDNF latency during the recovery period (30 min post-exercise) revealed a drastic decrease in plasma levels of BDNF (greater than 65%), remaining above the pre-exercise levels. In the present investigation, plasma levels of BDNF measured 30 min after the exercise also decreased significantly (curiously, around 65%), reaching, practically, the levels recorded before the intervention (Table 2). In another study, the authors observed a significant decrease in BDNF levels 30 min after intense exercise, falling below pre-exercise ones [19]. This short half-life of BDNF in the peripheral circulation has also been found in different studies [17,21,44], so it seems that the latency time of BDNF reactive to stress induced by exercise rarely exceeds 15 min. In any case, several factors such as exercise protocol (e.g., intensity, duration, recovery periods, and ergometer device), subjects’ characteristics (e.g., sex, age, healthy or patient, sedentary or active lifestyle), and biochemical assessment (e.g., salive, serum or plasma samples, assay method, and assay precision) could explain these differences in BDNF response.

On the other hand, and regarding WM capacity, pre-exercise DST scores (DST-F, DST-B and DST-T) coincide fully with those corresponding to the Spanish population of similar age range [46] (Figure 1). Moreover, all seems to indicate that high-intensity exercise stimulated WM capacity, improving DST performance by a short latency period. In fact, DST scores decreased slightly 30 min after exercise remaining above pre-exercise levels, which served to discard any practical effect.

As has been reported in previous studies, cognitive performance after a bout of acute exercise could be influenced by the intensity of exercise, which could be attributed to the secreted levels of biochemical markers (e.g., BDNF and CORT) or the states of arousal and neural activation [47]. Thus, physiological responses to exercise mediate changes in cognitive functioning through their direct effect on arousal status [48]. However, the level of arousal after exercise is strongly associated with the degree of exercise intensity with the proposed positive effects of a moderate arousal level on cognitive performance being based on the inverse-U theory. Accordingly, aerobic exercise of moderate intensity (60–80% VO_2max_ or HR_max_) is targeted as the most effective acute form of enhancing cognitive functioning [24,47]. The same theory has been used to explain possible associations between CORT and executive functioning (WM) so that moderate CORT levels being positively associated with executive functioning [33], while highly elevated CORT levels have been shown to interfere with the cognitive functions that are largely dependent on prefrontal networks (e.g., inhibitory control, attention regulation, and WM) [49]. However, and contrary to inverse–U theory and other studies which conclude that physical exercise can not be understood as a stimulus for WM [25], we demonstrated that HIIT can improve WM capacity in healthy young adults independently of the CORT response to intense exercise.

Nevertheless, and according to previous findings, both moderate-intensity exercise (MIE) and HIIT are capable of improving EF [26]. Moreover, in a recent study, female cancer survivors carried out both HIIT and MIE protocols; although there were no significant differences, HIIT produced moderate to large positive effects in comparison to MIE for outcomes including EF and WM [50].

As indicated previously, the high-intensity interval exercise used in our study was programmed to develop a cycling power equivalent to VO_2_ peak, a physiological parameter that is indicative of the high intensity of exercise. In addition, previous studies have shown an important CORT response when intermittent exercises were carried out [51,52], so the responses of CORT after a single bout of HIIT were to be expected (Table 2). Our results are consistent with those found by several authors who observed a huge increase in CORT levels 15 min after exhausting exercise [21,53]. In fact, and according to previous studies, the release of CORT can continue to increase until 30 min after performing an intense (anaerobic) exercise [54,55], which is coincident with our findings. The magnitude of the CORT responses to intense exercise assessed in our study (37% just at the end of the effort and 77% 30 min later) was also very similar to that measured previously by other authors, who found CORT increases of 50% during 30 min of recovery from high-intensity exercise [56].

Lastly, although no statistical relationships were found, remarkable BDNF and CORT responses were observed in the post-exercise situation. Thus, these increases in circulating levels of BDNF and CORT could be explained by their reactivity to exercise-induced stress and also by their supposed functions in the regulation of energy metabolism [57]. In any case, and contrary to previous findings [58], our results are in accordance with previous results which show significant increases in BDNF and CORT levels after intense exercise without any relationship between them [21]. On the other hand, the lack of a relationship between WM capacity and the responses of BDNF to intense exercise was also reported before [28]. In order to explain their results, these authors postulated that intense exercise could increase the arousal status and to enhance the attentional processes without a clear effect of BDNF on memory mechanism. In fact, a later study concluded that a single bout of intense exercise could increase the arousal status and neural activation, which further facilitate the central executive function related to the hippocampus and frontal lobe, two brain areas involved in WM [59].

Finally, it is necessary to consider some possible limitations in this study. First, a quasi-experimental design without a control group was adopted. This lack of control group could limit the external validity of the study findings. Second, the lack of diversity in the sample, which was composed only of physical education students. Third, studies with humans are limited to the assessment of circulating BDNF levels that may only provide an indirect association relative to central expression levels [29]. However, BDNF can reciprocally cross the blood–brain barrier, and it has been suggested that up to 70% to 80% of elevated peripheral levels of BDNF following acute exercise may be contributed by the brain [16]. On the other hand, although the main factors affecting the outcome measures were controlled by inclusion criteria, the presence of Val66Met polymorphism of BDNF gene in some of the participants could influence both their BDNF responses and WM capacity after exercise.

## 5. Conclusions

Overall, the main conclusion to be drawn from this study is that the stress induced by a single bout of HIIT induces a remarkable response of BDNF and CORT boosting of WM capacity in healthy young males. Moreover, the improvements in WM capacity are tangibles from just the end of intense exercise and they remain elevated up to 30 min after the effort had ended. Although we found a concurrence between BDNF and CORT responses with WM improvement, the lack of statistical associations between cognitive and neurobiological variables does not allow to us clarify the stimulating role of BDNF and CORT after intense exercise. This cognitive and biological connection might be addressed in future research which also should evaluate the potential effects of HIIT protocols on the remaining EF considering both healthy populations and patients with neurocognitive deficits.

## Figures and Tables

**Table 1:** Subjects’ characteristics and performance during the graded exercise test (session 1).

Variable	Mean ± SD
Age (years)	21.7 ± 2.1
Height (m)	1.77 ± 0.06
BMI (Kg/m^2^)	23.1 ± 1.4
Time to exhaustion (min)	13.6 ± 1.8
VO_2max_ (mL/kg/min)	47.1 ± 10.1
VT2 (mL/kg/min)	30.3 ± 9.9
VT2 (% VO_2max_)	64.6 ± 14.6
HR_max_ (bpm)	181.5 ± 8.4
VO_2_ peak (mL/kg/min)	45.3 ± 9.3
pVO_2_ peak (W)	275.0 ± 48.9

BMI = body mass index; VO_2max_ = maximal oxygen consumption; VT2 = second ventilatory threshold; HR_max_ = maximal heart rate; VO_2_ peak = peak oxygen consumption; pVO_2_ peak = cycling power corresponding; SD = standard deviation.

**Table 2:** Neurophysiological responses measured at the assessment time-points (session 2).

					Effect Size (*r*)		
	Pre Mean (SD)	Post Mean (SD)	Post 30 min Mean (SD)	Friedman Test (*p*)	Pre vs. Post	Post vs. Post 30 min	Pre vs. Post 30 min
BDNF (pg/mL)	424.66 (91.05)	1271.07 * (342.93)	437.04 (101.32)	<0.001	0.775	0.714	0.006
CORT (ng/mL)	132.33 (35.88)	181.41 *^‡^* (107.26)	234.45 *^†^* (109.63)	<0.001	0.716	0.425	0.828

BDNF = brain-derived neurotrophic factor; CORT = cortisol; pre = pre-exercise intervention; post = just at the end of exercise; post 30 min = 30 min aftere xercise. * p < 0.05 post vs. pre and post 30 min; ***^†^*** *p* < 0.05 post 30 min vs. pre and post; ***^‡^*** *p* < 0.05 post vs. pre (Wilcoxn signed rank test).

**Table 3:** Results of correlation analyses performed for BDNF and CORT across the assessment time-points.

	Pre—Post	Post—Post 30 min	Pre—Post 30 min
**BDNF**	0.768 ***	0.567 **	0.652 ***
**CORT**	0.645 ***	0.737 ***	-

BDNF = brain-derived neurotrophic factor; CORT = cortisol; Pre = before exercise; Post = just at the end of the exercise; Post 30 min = 30 min after high-intensity interval exercise. Data represents Spearman’s rho. ** *p* < 0.001; *** *p* < 0.001.
